# A systematic review of psychological difficulties among elite sports coaches

**DOI:** 10.3389/fpsyg.2025.1666035

**Published:** 2025-10-16

**Authors:** Sangwook Kang, Seungjoo Lee

**Affiliations:** Department of Physical Education, Seoul National University, Seoul, Republic of Korea

**Keywords:** occupational stress, burnout, mental health, coaching psychology, systematic review

## Abstract

**Introduction:**

This systematic review examined the psychological difficulties experienced by elite sports coaches, focusing on their causes, consequences, and potential interventions. Despite increasing recognition of the challenges faced by coaches, limited attention has been paid to understanding their psychological difficulties and available support systems.

**Methods:**

Following PRISMA guidelines, a comprehensive search was conducted across PubMed, Scopus, Web of Science, and SPORTDiscus. A total of 101 peer-reviewed studies published between 2010 and 2024 were identified and analyzed. Keywords such as “psychological difficulties," “stress," “burnout," and “coach-athlete relationship" were used to guide the search strategy.

**Results:**

Findings revealed that elite coaches frequently encounter psychological difficulties stemming from performance pressure, excessive workloads, the complexities of athlete management, and external social expectations. These stressors were associated with heightened risk of burnout, diminished psychological wellbeing, reduced coaching effectiveness, and strained coach-athlete relationships.

**Discussion:**

Despite the prevalence and severity of these psychological difficulties, relatively few studies have addressed intervention strategies specifically targeting coaches. This review highlights the urgent need for evidence-based support systems to help coaches manage psychological difficulties and optimize their professional functioning. The findings provide valuable insights to inform future research and practical initiatives aimed at creating a healthier and more sustainable environment for both coaches and athletes in elite sports settings.

## 1 Introduction

In the context of modern sport, coaches have come to be recognized as more than mere instructors of technical and tactical skills. They are increasingly acknowledged as pivotal figures who significantly influence not only athletic performance, but also athletes' psychological wellbeing and overall quality of life (Felton and Jowett, 2013; Mageau and Vallerand, 2003; Reinboth et al., 2004). For instance, positive coaching behaviors have been shown to foster athletes' intrinsic motivation, self-efficacy, and team cohesion (Mageau and Vallerand, 2003; Reinboth et al., 2004). Autonomy-supportive behaviors and positive feedback from coaches are also associated with the satisfaction of athletes' basic psychological needs, thereby enhancing their overall psychological wellbeing (Felton and Jowett, 2013).

Conversely, authoritarian or controlling coaching styles have been linked to reduced motivation and increased risk of athlete burnout (Rocchi and Pelletier, 2017), and in more extreme cases, have resulted in verbal or emotional abuse with long-term consequences for athletes' mental health (Stirling and Kerr, 2013). Such findings underscore the significant role of coaching behavior in shaping athletes' performance and development, and importantly, highlight its close relationship with coaches' own psychological functioning (Thelwell et al., 2008, 2017; Olusoga et al., 2012). Thus, investigating coaches' psychological states and exploring effective intervention strategies is essential—not only for promoting athlete wellbeing, but also for safeguarding the health of the coaches themselves.

In contemporary elite sport, the demands and pressures placed upon coaches are greater than ever before. Expectations from team owners, sponsors, media, and fans, alongside the pressure to win, place an immense psychological burden on coaches (Olusoga et al., 2012; Thelwell et al., 2017). In highly competitive environments such as professional leagues or national teams, a single defeat can jeopardize a coach's career. As a result, many coaches are subjected to chronic stress, excessive workloads, performance pressure, and challenges in maintaining work-life balance, all of which contribute to elevated stress levels (Fletcher and Scott, 2010; Norris et al., 2017).

Prolonged exposure to such stress can severely impair both physical and mental health. Empirical evidence indicates that many coaches suffer from insomnia, gastrointestinal issues, headaches, depression, anxiety, and difficulties with anger management (Bissett and Tamminen, 2022; Altfeld et al., 2015). These health issues not only diminish quality of life but also hinder job performance. For instance, reduced concentration and impaired decision-making under stress can lead to strategic errors during crucial competitions—potentially compromising team success.

Traditionally, sport psychology has focused on enhancing athletic performance and promoting athlete wellbeing through interventions such as counseling, psychological skills training (PST), and self-regulation strategies. While most of this literature has centered on how coaches influence athletes, comparatively little research has examined the psychological health of coaches themselves or sought to identify means of improving it. However, a recent systematic review by Norris et al. (2017) comprehensively analyzed stressors, coping mechanisms, and wellbeing among sports coaches. Their findings indicate that although interest in the psychological health of coaches is growing, considerable gaps in the literature remain.

Against this backdrop, the present study aims to conduct a comprehensive review of the existing literature on the psychological challenges faced by elite sport coaches. Through a systematic review methodology, this research seeks to identify key trends, methodologies, and findings, while also highlighting limitations and proposing future research directions. The results are expected to provide a foundational understanding for the development of practical strategies to promote coach wellbeing in elite sport settings. Ultimately, such efforts will contribute to the creation of a healthier sporting environment that supports the growth and development of both coaches and athletes. Specifically, this study aims to address the following research questions:

1. What are the main causes and consequences of coaches' psychological difficulties identified in previous studies?
2. What intervention methods have been proposed to promote coaches' psychological health, and how is their effectiveness being evaluated?
3. What are the methodological and theoretical limitations of existing studies, and how are future research directions being presented?

## 2 Methods

This study utilized a systematic literature review method to identify research trends related to coaches' psychological difficulties, following the PRISMA guidelines (Moher et al., 2009). Specifically, the process included: (1) formulating research questions, (2) setting inclusion and exclusion criteria, (3) database search, (4) literature selection, (5) data extraction, and (6) result synthesis.

### 2.1 Design

A comprehensive literature search was conducted across four major academic databases: PubMed, Scopus, Web of Science, and SPORTDiscus. The search was limited to peer-reviewed articles published between 2010 and 2024, and employed keywords such as “*psychological difficulties”*, “*stress”*, “*pressure”*, “*burnout”*, “*coach mental health”*, “*coach-athlete relationship”*, and “*coach burnout”*. These terms were applied individually, and were not required to co-occur within a single study. Only sport-specific research focusing on coaches was included, while studies from general psychology, education, or medicine unrelated to sport were excluded.

During the literature selection process, the following inclusion and exclusion criteria were applied: (1) Both quantitative and qualitative research designs were considered. (2) Studies had to focus on coaches working with elite athletes. (3) Eligible studies examined coaches' psychological difficulties, including the underlying causes and psychological consequences. (4) Studies conducted from the athlete's perspective—for example, research investigating the impact of coach stress on athletes—were excluded.

For the purpose of this review, “elite sport” was defined as high-performance competitive sport, including professional leagues, national teams, and athletes regularly competing at national or international championships. Coaches operating in these settings were categorized as elite sport coaches, given the unique psychological demands and occupational pressures associated with such environments.

## 3 Results

### 3.1 Research on coach burnout

Research related to coach burnout accounted for 40 studies, representing 37% of the total. Olusoga et al. (2019) comprehensively reviewed literature related to sports coach burnout, analyzing research trends and limitations, and suggesting future research directions. This study identified 65 publications from 1984 to September 2017 and analyzed 45 research papers among them. According to the main results, the studies mainly targeted coaches in North America and Europe, and coach burnout was studied from perspectives such as stress, Self-Determination Theory (SDT), and Work-Home Interference.

The main variables predicting coach burnout included excessive workload, lack of recovery, and work-home imbalance. Also, there were considerable differences in the scales and methods used to measure burnout across studies, with the Maslach Burnout Inventory (MBI) being mainly used, but often in different versions or modified forms across studies. Some studies measured both the intensity and frequency of burnout, while others evaluated burnout by measuring only emotional exhaustion.

In conclusion, while research on coach burnout is increasing, there has been a strong reliance on quantitative, cross-sectional study designs, which are less suited to capturing the enduring and evolving nature of burnout experiences. Indeed, Olusoga et al. (2019) note that although such studies are useful for broadening our understanding, they “do little to reflect the ‘enduring' burnout experience,” pointing to a notable lack of longitudinal research. Supporting this critique, Altfeld et al. (2015) conducted a longitudinal study tracking coaches' burnout, stress, and recovery across a competitive season, demonstrating temporal fluctuations in these variables that cross-sectional designs could not adequately detect.

### 3.2 Research on coach stress

A total of 32 studies, accounting for 31.7%, were found to be related to coaches' stress. In the sports environment, coaches perform various roles and must meet several expectations, so they can be classified as an occupation with high stress levels. Coaches‘ stress and resulting wellbeing greatly affect not only their performance but also the performance of the athletes they guide. Therefore, understanding the stress experienced by coaches and their impact on wellbeing plays an important role in creating a positive sports environment and optimizing the performance of both coaches and athletes.

**Figure 1:**
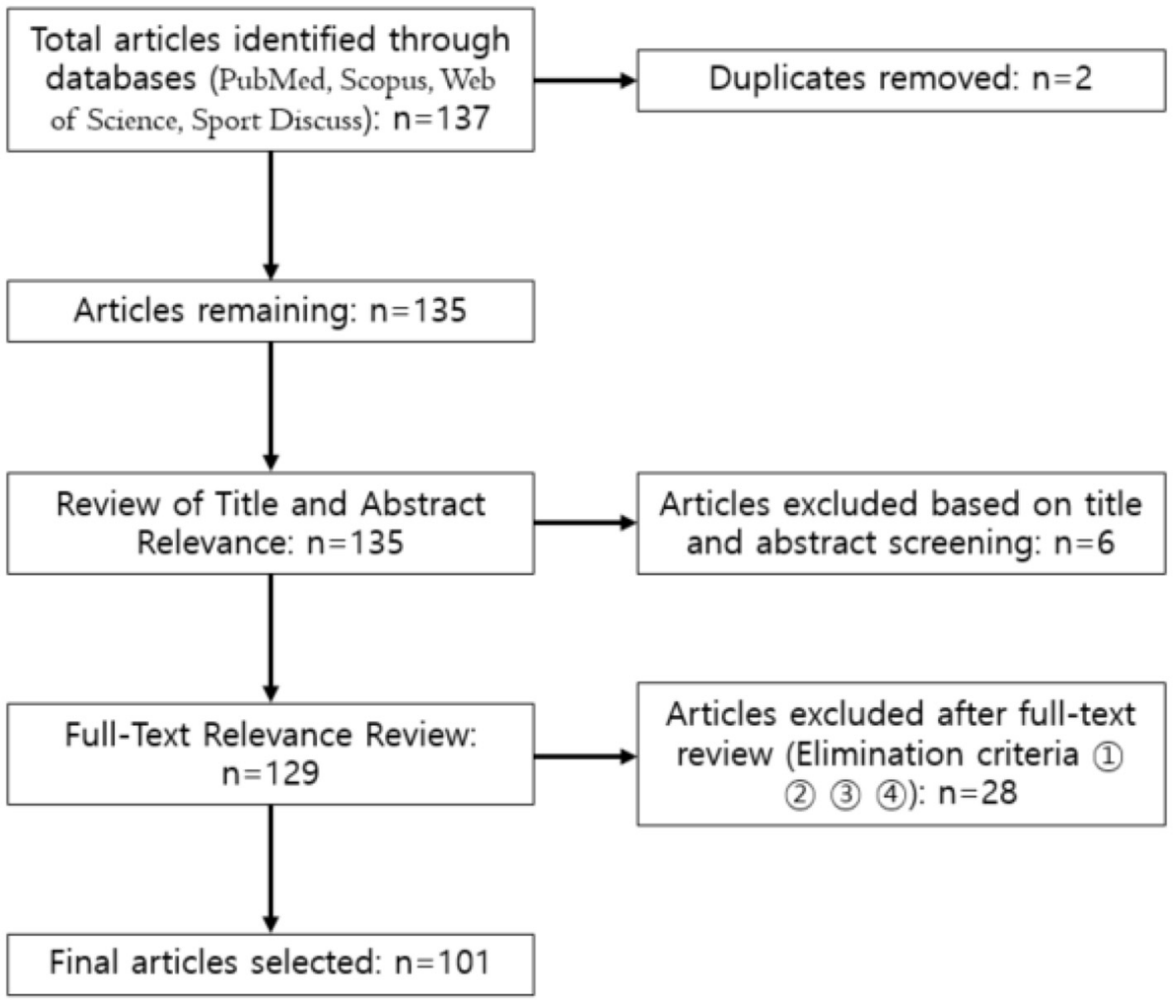
Literature search process. Elimination criteria. 1. Included both quantitative and qualitative studies. 2. Studies on non-elite coaches. 3. Studies not addressing coaches' psychological difficulties. 4. Studies not from the coach's perspective.

The stress factors faced by sports coaches are diverse and complex. Major stressors include relationships with athletes, work-related responsibilities, time pressure, performance pressure, burden of game results, and the high expectations set by themselves (Bentzen et al., 2016; Fletcher and Scott, 2010; Olusoga et al., 2009). Also, organizational factors can be sources of stress, including training environment, financial pressure, team composition and internal conflicts, administrative issues, and external demands such as travel (Fletcher and Scott, 2010). When facing these stress factors, coaches show cognitive, emotional, physiological and behavioral reactions. These reactions ultimately affect coaches' performance and psychological state, and the details of each reaction are shown in Figure 1. If such stress accumulates over the long term, coaches can reach burnout. Burnout manifests as emotional exhaustion reduced personal accomplishment, and depersonalization, and coaches working in elite sports environments experience burnout more frequently. This can have serious effects not only on coaching performance but also on personal life.

Therefore, future research should not only examine the various stress factors faced by coaches but also conduct empirical studies on intervention methods that can alleviate this stress. Additionally, there is a need to strengthen support systems at the organizational level so that coaches can work in a healthier environment. To this end, the introduction of educational programs for building positive relationships between coaches and athletes should also be considered. Through this, coaches can maintain high performance while maintaining psychological wellbeing and prevent burnout in the long term.

### 3.3 Research on coach mental health

Research on coaches' mental health comprised 5 studies, representing 5% of the total. Mental health research in elite sports has mainly focused on athletes‘ mental health, but relatively less attention has been paid to important sports occupational groups such as coaches (Breslin et al., 2022). However, coaches, like athletes, are vulnerable to mental health issues such as stress, anxiety, and depression (Carson et al., 2018). Recent evidence also highlights that elite-level coaches report considerable prevalence rates of mental health symptoms, with stressors related to performance pressure, workload, and interpersonal relationships being particularly influential (Kegelaers et al., 2021). If these issues are not properly prevented and managed, coaches' job performance can deteriorate and develop into long-term health problems. Therefore, it is very important to detect mental health problems early and intervene appropriately. Especially, given the significant role of coaches in elite sports environments, research on coaches‘ mental health needs to be actively conducted.

The culture that emphasizes mental toughness in the sports environment can make not only athletes but also coaches hesitant to seek help for mental health issues. However, Breslin et al. (2022) show that when a culture of raising awareness and actively managing mental health issues is fostered, both coaches and athletes can deal with mental health issues more openly. Therefore, mental health education and support programs for coaches are needed. In particular, resilience has been shown to buffer the negative impact of stressors on coaches' mental health (Kegelaers et al., 2021), suggesting that interventions designed to strengthen resilience could be especially effective. These programs can help coaches maintain their own mental health while providing the skills and knowledge needed to support athletes‘ mental health. For this, more systematic approaches need to be developed through research and policy support.

### 3.4 Psychological aspects of coaches and coach-athlete relationships

This line of research comprised only 5 studies, accounting for ~5% of the total. This reflects the fact that, unlike most existing research on the coach–athlete relationship that has predominantly focused on athletes' perspectives, relatively few studies have centered on coaches' psychological aspects or their roles. The coach-athlete relationship is considered a very important element in sports psychology, and many studies consistently emphasize that the relationship between coaches and athletes has a significant impact on athletes' performance, motivation, and psychological state (Jowett and Cockerill, 2003; Jowett and Lavallee, 2007; Jowett and Ntoumanis, 2004). One of the important factors determining the quality of the coach-athlete relationship is the psychological state of the coach. According to Jowett and Cockerill (2003), when coaches maintain a positive psychological state, effective communication with athletes becomes possible, making athletes feel greater trust and bond. On the other hand, when coaches experience stress or anxiety, changes occur in non-verbal behavior or verbal communication, which can cause confusion for athletes and deteriorate the coach-athlete relationship.

Jowett and Lavallee (2007) also emphasized that psychological management of coaches can improve coach-athlete relationships and positively affect athletes. These studies suggest the need for systematic programs to manage and support coaches' psychological health and imply the need for in-depth exploration of antecedent factors that can affect coach-athlete relationships. McShan and Moore's (2023) study aimed to systematically review variables related to coach-athlete relationships from coaches' perspectives and analyzed 57 studies published from 2000 to 2021. According to the main results, the higher coaches felt their job satisfaction, the more positive association they showed with sub-factors of coach-athlete relationships such as closeness, commitment, and complementarity in relationships with athletes. On the other hand, the higher the level of coaches' burnout or stress, the more negatively the coach-athlete relationship was affected (Thelwell et al., 2017). Also, coaches' conflict management ability was found to have a significant impact on coach-athlete relationships, and if coaches fail to effectively manage conflicts with athletes, relationships deteriorate, which negatively affect athletes' growth and performance (Jowett, 2003).

These research results show that the quality of coach-athlete relationships is largely determined by coaches' job satisfaction, burnout, stress management, and conflict resolution abilities. Therefore, it is important to create an environment where coaches can enjoy their jobs and appropriately manage stress. Also, education and support are needed for coaches to improve their conflict resolution abilities and build positive relationships with athletes based on trust and respect. This will ultimately have a positive impact on athletes' growth and performance and contribute to maintaining healthy coach-athlete relationships in the long term.

### 3.5 Intervention research for psychological aspects of elite sports coaches

In competitive sports environments, coaches are continuously exposed to diverse stress factors such as performance pressure, media scrutiny, interpersonal conflicts with athletes and staff, and organizational demands (Thelwell et al., 2016; Bentzen et al., 2016). These stressors can lead to burnout, diminished wellbeing, and impaired decision-making, ultimately influencing athletes' performance and the overall team climate. Although the importance of psychological support for coaches has been frequently emphasized, empirical research on interventions specifically targeting coaches remains scarce compared to the substantial body of work focusing on athletes. Preliminary findings from intervention studies (e.g., Olusoga et al., 2014; Didymus and Fletcher, 2017) suggest that psychological skills training and stress management programs can enhance coaches' coping resources and resilience. Given that coaches' psychological states directly shape athletes' motivation, confidence, and performance, sustained intervention research targeting coaches is vital. Such research would not only safeguard coaches' psychological health but also contribute to the optimal performance and wellbeing of athletes under their guidance.

#### 3.5.1 Mindfulness

In the study by Longshore and Sachs (2015), the effects of mindfulness training on anxiety reduction, emotional stability improvement, and stress management were examined for NCAA division coaches. Through 6 weeks of mindfulness training, coaches‘ anxiety levels decreased, emotional stability improved, and the balance between positive and negative emotions improved. Additionally, coaches reported improved self-awareness and emotion regulation abilities after mindfulness training, which had a positive impact not only on relationships with athletes but also on personal life. This study suggests that mindfulness training can be an important intervention method for improving coaches' psychological wellbeing and presents new possibilities for sports psychology interventions targeting coaches.

#### 3.5.2 Psychological skill training (PST)

In the study by Olusoga et al. (2014), MST was applied to sports coaches at a British university to verify the effectiveness of the program. Olusoga and colleagues point out that while existing psychological skills training has been applied to athletes, there is a lack of programs for coaches who have difficulty managing stress in competitive sports environments.

In this study, a 6-week MST program was conducted with five British elite sports coaches. As a result, coaches showed significant improvements in confidence and relaxation skills and reported positive changes such as decreased anxiety levels and reduced use of negative coping strategies (e.g., self-blame). While most psychological skills training studies have focused on athletes, this study shows that coaches can also use psychological skills to improve their coaching abilities in stressful situations. Furthermore, this study is significant in that it views coaches as ‘performers', emphasizes that coaches also need psychological preparation, and provides strong evidence that MST should be included in educational programs for coaches.

The psychological difficulties experienced by coaches can directly affect not only the coaches themselves but also the athletes' performance and psychological state. Therefore, it is urgent to develop systematic sports psychology counseling programs to improve the psychological aspects of coaches, and various research and interventions to support coaches' psychological state are needed. These programs and research can ultimately maximize the performance of both coaches and athletes and contribute to creating a healthier sports environment.

### 3.6 Methodological limitations in elite coach research

While previous studies primarily examined the influence of elite coaches' psychological aspects on athletes, more recent research has begun to focus on coaches themselves, exploring their complex roles, work environments, and psychological interventions aimed at enhancing their psychological wellbeing. Nevertheless, research directly addressing coaches remains limited, and several methodological shortcomings have been identified. First, because elite coaches represent a highly specialized group, it is often difficult to recruit sufficiently large samples for research. Bentzen et al. (2016) pointed out that studies on elite coaches often suffer from small sample sizes, limiting the generalizability of findings, while Gearity (2012) also emphasized that restricted sample sizes can undermine the reliability of results. Furthermore, when research targets coaches from specific sports only, the findings may not easily apply to other contexts, adding another layer of limitation. Second, many studies employ qualitative methods to capture coaches' experiences and perceptions in depth, which are valuable for understanding subjective realities but are vulnerable to the influence of researcher subjectivity. Cushion (2010) noted that because interpretation plays a critical role in qualitative analysis, the results may be shaped by the researcher's perspective, restricting their universal applicability. Third, self-report questionnaires are commonly used to assess elite coaches' psychological states and stress levels. While self-reports can provide meaningful insights, their accuracy depends heavily on respondents' self-awareness and honesty. In particular, elite coaches may deliberately underreport psychological difficulties to preserve authority or maintain a professional image, which can create a gap between reported and actual psychological states (Fletcher and Scott, 2010). Fourth, the predominance of cross-sectional research designs represents another limitation. Such studies capture coaches' psychological states at a single point in time, which fails to reflect the dynamic nature of stress and psychological challenges that evolve over the course of a season or career. Longitudinal studies are therefore crucial; Raedeke (2004), for instance, conducted a 1-year follow-up study on coach burnout, demonstrating how stress and burnout can develop and change over time, thereby highlighting the importance of tracking these processes systematically. Fifth, research has often neglected gender and cultural diversity. In male-dominated elite sports environments, the experiences of female coaches may not be adequately represented, and the contextual challenges faced by coaches from minority cultural backgrounds may remain overlooked. Norman (2010) argued that there is still a lack of research focusing on female and minority-culture coaches, with most studies centered on male elite coaches, thereby limiting the applicability of findings across genders and cultural contexts. Taken together, these methodological limitations—including small sample sizes, the subjectivity of qualitative research, the reliability concerns of self-report data, the scarcity of longitudinal studies, and the insufficient consideration of gender and cultural diversity—restrict the reliability and generalizability of findings.

## 4 Discussion

This study aimed to identify current research trends and suggest future research directions through a systematic literature review on the psychological difficulties experienced by elite sports coaches. The analysis results showed that coaches' psychological difficulties arise from various factors and have a significant impact not only on the coach's individual wellbeing but also on athletes' performance and team outcomes. In this chapter, we will develop an in-depth discussion based on the main research results.

### 4.1 Multidimensional characteristics and performance implications of coaches' psychological difficulties

The psychological difficulties experienced by coaches are multidimensional rather than stemming from a single factor. Consistent with Fletcher and Scott (2010), stressors can be broadly categorized into personal, organizational, and sociocultural domains. At the personal level, issues such as performance pressure, self-doubt, and work–life balance difficulties emerge; at the organizational level, excessive workload, role conflict, and lack of resources are prevalent; and at the sociocultural level, a win-at-all-costs culture, media scrutiny, and job insecurity act as significant stressors. For instance, Olusoga et al. (2012) found that Olympic coaches faced stress not only from competition outcomes but also from athlete management, organizational politics, and media demands, highlighting the multifaceted nature of these difficulties.

These stressors also have complex implications for coaches' performance. While moderate stress can enhance focus and motivation (Thelwell et al., 2017), excessive stress impairs decision-making, emotion regulation, and communication, thereby diminishing coaching effectiveness. Kellmann et al. (2016) further demonstrated that highly stressed coaches exhibit cognitive impairments such as reduced attention and memory, emotional difficulties including anxiety and depressive symptoms, and behavioral changes such as rigid leadership styles and maladaptive communication patterns. Such findings underscore the importance of adopting an integrated, multi-layered approach to managing coaches' psychological difficulties—encompassing not only individual interventions but also organizational reforms and shifts in societal perceptions.

Beyond individual performance, coaches' psychological wellbeing significantly shapes the quality of coach–athlete relationships. As Jowett and Cockerill (2003) emphasized, these relationships are grounded in trust, respect, and communication; yet when coaches experience heightened stress, these foundations can be compromised. Stressed coaches may adopt authoritarian or inconsistent communication, eroding athletes' trust and respect, while negative emotions can be transmitted to athletes through emotional contagion (Davis et al., 2018). Expanding on this, Jowett's (2025) 25-year review highlighted how psychological strain and burnout erode the 3+1Cs of relationship quality—closeness, commitment, complementarity, and co-orientation. Similarly, a systematic review by McShan and Moore (2023) showed that stress and burnout consistently undermined relationship quality, whereas coaching satisfaction, harmonious passion, and the fulfillment of basic psychological needs supported stronger bonds. Importantly, autonomy-supportive and caring coaching behaviors were associated with higher-quality relationships, while controlling or autocratic behaviors weakened them.

Nevertheless, research explicitly examining the coach–athlete relationship from the coaches' perspective remains limited. Most studies continue to privilege athletes' accounts, leaving an incomplete picture of relational dynamics (McShan and Moore, 2023). Taken together, these findings indicate that coaches' psychological wellbeing is not only central to their own performance but also a critical determinant of coach–athlete relationship quality. Supporting coaches in managing stress and maintaining psychological health is therefore essential not only for their personal wellbeing but also for safeguarding relational quality, thereby promoting athletes' motivation, performance, and long-term positive sport experiences.

### 4.2 Effectiveness of psychological difficulty management strategies

The literature reviewed in this study reports on the effectiveness of various strategies for managing coaches‘ psychological difficulties. Particularly noteworthy are cognitive-behavioral approaches, mindfulness-based interventions, and the utilization of social support networks. Regarding cognitive-behavioral approaches, Olusoga et al. (2014) showed that Psychological Skills Training (PST) is effective in managing coaches' stress and improving performance. In this study, coaches who participated in the MST program reported improved confidence, reduced anxiety, and enhanced ability to cope with stressful situations. This suggests that psychological skills training, traditionally applied to athletes, can also be usefully utilized for coaches.

In the case of mindfulness-based interventions, the study by Longshore and Sachs (2015) is noteworthy. In their study, coaches who participated in a 6-week mindfulness training program experienced stress reduction, improved emotional stability, and increased attention. It was particularly emphasized that mindfulness training improves coaches' emotional awareness and regulation abilities, enabling them to respond more effectively in stressful situations. The importance of social support networks was consistently evident in several studies. For example, according to the systematic literature review by Norris et al. (2017), forming networks with fellow coaches, mentoring systems, and organizational-level support play a crucial role in managing coaches' stress. This suggests that managing coaches' psychological difficulties is insufficient with individual effort alone and requires support systems at the organizational and community levels.

It is also worth noting that most counseling-related studies in sport psychology have primarily emphasized the role of coaches as supporters or facilitators of athletes' psychological wellbeing (Bissett et al., 2020; Simons and Bird, 2023). While this perspective highlights the importance of coaches in athlete-centered interventions, it has left a significant gap in addressing the counseling and psychological support needs of coaches themselves. Indeed, research directly targeting counseling or structured psychological interventions for coaches remains almost non-existent. Notable exceptions include behavioral training to enhance need-supportive coaching styles (Reynders et al., 2019) and large-scale educational initiatives such as the Million Coaches Challenge (MCC), which aims to improve mental health awareness among youth coaches (Parents, 2024). Furthermore, models from digital cognitive-behavioral interventions and integrative coaching psychology (Fitzsimmons-Craft et al., 2023; Pahlavnejad et al., 2024) provide valuable frameworks for developing future coach-centric counseling programs. This gap underscores the urgent need for research on tailored, evidence-based counseling interventions specifically designed to support coaches' psychological wellbeing.

### 4.3 Methodological limitations in research on elite coaches

Research on elite coaches faces several methodological limitations that can impact the reliability and generalizability of findings. First, qualitative research methods are commonly used to explore elite coaches' experiences and perceptions in depth, but they are subject to research bias. Since qualitative analysis relies heavily on interpretation, researchers' subjectivity can influence results, limiting their applicability to different coaching contexts (Cushion, 2010). Second, self-report questionnaires, frequently used to assess elite coaches' psychological states and stress levels, present reliability concerns. While self-reported data offers valuable insights, it is dependent on respondents' self-awareness and honesty, which can introduce bias. Elite coaches may underreport their psychological difficulties to maintain their authority and professional image, leading to discrepancies between reported and actual psychological states (Fletcher and Scott, 2010). Third, the lack of longitudinal studies limits our understanding of the long-term psychological challenges faced by elite coaches. Research on coaches has frequently relied on cross-sectional designs rather than longitudinal approaches. However, since coaches' stress levels and experiences evolve over time, it is crucial to track these changes systematically. Raedeke (2004) demonstrated the importance of longitudinal research by conducting a 1-year follow-up study on coach burnout, highlighting how stress and burnout develop over time. To improve the robustness of research in this field, future studies should enhance methodological rigor, incorporate objective measures, and adopt longitudinal approaches to provide a more comprehensive understanding of elite coaches' psychological challenges.

Issues with measurement tools can also be pointed out. Many studies were using general stress scales or burnout scales, which may have limitations in accurately measuring coach-specific psychological difficulties. It seems that more development and validation of coach-specific tools, such as the Coach Burnout Questionnaire developed by Raedeke and Smith (2001), is needed. This methodological review raises the need for more diverse and sophisticated research designs and measurement methods in future research on coaches' psychological difficulties. If longitudinal designs, mixed research methods, and the development of coach-specific measurement tools are implemented, it seems possible to understand coaches' psychological difficulties more deeply.

## 5 Summary and suggestions

This study identified current research trends and suggested future research directions through a systematic literature review on the psychological difficulties of elite sports coaches. The findings confirmed that coaches' psychological difficulties are multidimensional in nature and represent an important factor that significantly impacts not only coaches' individual wellbeing but also athletes' performance and team outcomes.

To effectively address these challenges, an integrated approach at personal, organizational, and sociocultural levels is required. At the personal level, cognitive-behavioral approaches and mindfulness-based interventions may be effective, while at the organizational level, the establishment of coach support systems and mentoring programs could be beneficial. At the sociocultural level, efforts should be made to challenge the prevailing win-at-all-cost culture and to increase public awareness of the vital role of coaches.

Nevertheless, this review is not without limitations. First, the majority of the included studies were conducted in Western cultural contexts, which may restrict the generalizability of the findings to non-Western settings. Second, by focusing primarily on elite sports coaches, the review did not fully capture the experiences of coaches operating at other competitive levels, such as youth, amateur, or recreational sport. Third, the possibility of publication bias cannot be ruled out, as studies reporting significant results are more likely to be published than those with non-significant findings. These limitations highlight the need for future research to broaden the scope of investigation by incorporating studies from more diverse cultural contexts and across multiple levels of coaching, as well as by considering unpublished or gray literature to minimize the risk of publication bias.

Recommendations for future research on coaches' psychological difficulties are as follows. First, longitudinal research designs are essential for understanding how coaches' psychological difficulties evolve over time and what patterns they exhibit. Psychological challenges faced by coaches are not one-time occurrences; rather, stressors arising at a given moment may accumulate or recur, thereby intensifying over time. To capture these processes, long-term follow-up studies are indispensable. For example, stress, anxiety, and burnout experienced by coaches during a competitive season may fluctuate depending on the stage of the season, while external factors such as competition outcomes, relationships with athletes, and organizational expectations may also shift over time. Longitudinal research can thus provide insights into the dynamic interplay of these factors and their impact on coaches' psychological wellbeing that cross-sectional studies cannot fully reveal.

Second, the development and evaluation of psychological intervention programs for coaches represent a critical task in addressing the various psychological challenges they face. Coaches are vulnerable to stress, burnout, anxiety, and psychological fatigue, yet systematic intervention programs to manage and improve these issues remain limited. Therefore, research on both the development and effectiveness of such interventions is necessary. Importantly, programs should adopt a tailored approach that reflects the environmental factors and roles specific to coaches. Psychological difficulties may stem from multiple sources, including athletes' performance, team outcomes, organizational pressures, and personal stressors. Taking these into account, programs can be designed by integrating approaches such as mindfulness-based interventions (MBIs), cognitive-behavioral therapy (CBT), and emotion regulation training. Each of these methods can offer differentiated strategies according to the specific psychological difficulties faced by coaches and may provide more personalized solutions.

Third, psychological interventions must also be responsive to environmental changes. The global outbreak of COVID-19 significantly affected not only the broader sports environment but also the coaching context. According to Stambulova et al. (2022), coaches experienced novel forms of psychological stress resulting from heightened uncertainty, shifts in training methods, and changes in relationships with athletes under new environmental demands. In particular, goal readjustments due to cancellations or postponements of competitions, adaptation to online coaching, and concerns about personal and athlete health represented unprecedented challenges not sufficiently addressed in previous research. These findings highlight the need to incorporate strategies for coping with environmental changes when studying coach stress and adaptation. Accordingly, future support programs for coaches should include competencies such as managing uncertainty in crisis situations, developing digital coaching skills, maintaining and restoring relationships, and addressing health-related anxiety. Psychological support that enhances coaches' ability to flexibly adapt to rapidly changing environments will be crucial not only for their personal wellbeing but also for sustaining coach–athlete relationships and team performance.

Fourth, there is a need to evaluate the long-term effects of psychological intervention programs. Even when short-term interventions appear effective, their benefits may diminish over time or potentially produce unintended negative consequences. Thus, longitudinal evaluations of intervention effectiveness are necessary to confirm their sustainability and to identify areas for improvement and refinement.

In conclusion, this study provides a comprehensive understanding of the psychological difficulties faced by elite sports coaches and underscores the need for multifaceted approaches to address them. Building upon these research directions, future in-depth studies may contribute to enhancing coaches' psychological wellbeing, which in turn can promote athletes' performance and team outcomes. Ultimately, such efforts will support the healthy development of sport as a whole and foster sustainable performance at both individual and organizational levels.

## Data Availability

The raw data supporting the conclusions of this article will be made available by the authors, without undue reservation.
